# Separation of Inorganic Forms of Tellurium Using On-Site SPE Followed by ICP-MS or ICP-OES—The Right Solution for Water Monitoring

**DOI:** 10.3390/molecules30020303

**Published:** 2025-01-14

**Authors:** Katarzyna Kińska, Barbara Żelazko, Olga Gajewska, Magdalena Borowska, Monika Sadowska, Beata Krasnodębska-Ostręga

**Affiliations:** 1 Faculty of Chemistry, University of Warsaw, ul. Pasteura 1, 02-093 Warsaw, Poland; kkinska@chem.uw.edu.pl (K.K.); b.zelazko@student.uw.edu.pl (B.Ż.); o.gajewska@student.uw.edu.pl (O.G.); msadowska@chem.uw.edu.pl (M.S.); 2 Faculty of Chemistry, Warsaw University of Technology, ul. Noakowskiego 3, 00-664 Warsaw, Poland; magdalena.borowska@pw.edu.pl

**Keywords:** tellurium speciation, ICP-MS, HG-ICP-OES, on-site sample pretreatment, solid phase extraction, Te(IV), Te(VI)

## Abstract

Tellurium, recognized as one of the technology-critical elements, should be considered as a xenobiotic. Its application, i.a. in the growing photovoltaic industry, raises concerns about Te(IV) and Te(VI) release to the environment. As both forms differ in mobility and toxicity, Te speciation should be included in water monitoring, but problems with speciation changes occurring during sampling, transport, and sample storage require the use of on-site separation of Te forms. A simple procedure based on solid phase extraction (SPE) with the anionic exchange mechanism (SAX, involving commercially available columns), followed by their quantification with elemental techniques, has a high potential for implementation in routine analysis. The proposed SPE-ICP-MS (inductively coupled plasma mass spectrometry) method allows direct analysis of Te(VI) and Te(IV), with Te(IV) determined after elution from the column. The detection limits obtained for the 5.0 mL sample are 0.02 ng mL^−1^ and 0.05 ng mL^−1^ for Te(VI) and Te(IV), respectively. Hydride generation inductively coupled plasma optical emission spectrometry (HG-ICP-OES) was used to control possible changes in tellurium speciation occurring during species isolation using SPE. The simple and fast water pretreatment proposed here offers the possibility of separating Te(IV) and Te(VI) at the sampling site, and the elution of Te(IV) does not have to be conducted on-site. Application to the river water and seawater matrix proved the feasibility of incorporating Te speciation analysis into routine water analysis.

## 1. Introduction

Tellurium is a relatively rare element, with concentration in the Earth’s crust estimated to be only 1–5 μg kg^−1^ according to various sources [1]. It is obtained mainly from copper anode slimes and from residues obtained after smelting processes of metals (Ni, Pb, Zn, precious metals). Te is a metalloid belonging to technology-critical elements (European COST Action TD1407: Network on Technology Critical Elements—TCEs) [2,3], for which the demand has increased due to their application in emerging technologies, and which are characterized by a rather scarce supply. Solar panel production accounts for 60% of the annual Te consumption, thermoelectric production—20%, and metallurgy—10% [4].

Similarly to many trace elements from the TCE group, initially the impact of Te on living organisms has not been much of a concern due to low concentrations and lack of a meaningful industrial role in the past. This is gradually changing, as the process of extracting, manufacturing, and scrapping tellurium products results in the release of large quantities of tellurium ions into aquatic systems [5]. Thus, Te is becoming more and more frequently mentioned as a new xenobiotic. An often-raised issue is also the potential risk to human health and the environment associated with the use of large quantities of CdTe photovoltaic panels (PVs)—labelled as “green” and environmentally friendly energy. The production waste and used PVs are simply deposed of in municipal landfills. This poses serious health and environmental hazards as their components may leach out of the landfills and contaminate soil, groundwater, or surface water [6]. As CdTe is unlikely to be released from PV modules during use or disposal, concerns focus more on other Cd and Te species, rather than CdTe itself. In particular, there is rising concern about Te species leaching from landfills after inadequate disposal of CdTe thin film PVs [7].

The chemistry of Te depends on its oxidation state. Environmentally, the most relevant forms are those at −2, 0, +4 and +6, the presence of which is influenced by redox conditions and pH [8]. Tellurium in non-polluted surface waters occurs in small amounts of 20 ng L^−1^, which is caused by the strong adsorption of its oxyanions to mineral surfaces. Concentrations of total dissolved tellurium in open oceans are usually less than 2 ng L^−1^ [9]. Tellurium in water mostly occurs as Te(IV) and Te(VI) oxyanions [1], and both are considered to be toxic. Their toxicity depends on speciation—tellurates(IV) are about ten times more harmful than tellurates(VI), which are already more toxic than selenates(IV) and arsenates(III) [10]. It should be emphasized that the anions are highly soluble and mobile in the environment. Tellurium oxyanions present in the water system have damaging effects on both prokaryotic and eukaryotic organisms, and low concentrations of Te(IV) increase the oxidative stress [11]. Once the tellurium concentration in drinking water exceeds 2 mg L^−1^, it poses a considerable threat to human health [5]. Some authors have reported that tellurium pollution could be linked to the occurrence of autoimmune, neurodegenerative, and oncological disorders [11]. Therefore, the study of tellurium, including speciation analysis, should be considered in modern monitoring of water reservoirs as part of the sustainable development of new technologies. In particular, reports about the presence of Te anions in lake water [12] and seawater [13] have already been published.

An important aspect in the mobility of forms of Te in water systems is the (in)stability of Te anions. The equilibrium between various forms depends on the chemical condition of the environment, and the dependencies might be complicated. Therefore, the sole act of sampling can disturb the preliminary speciation ratio between Te(IV) and Te(VI) because changes in temperature, oxygenation, and/or exposure to light are able to change the speciation [14]. Metalloid oxyanions could be oxidized in the presence of Fe and Mn oxides (suspended particles in the water matrix) as a result of photocatalysis (homo- or heterogenic) occurring under UV-Vis exposure. Photooxidation is promoted under a specific pH. The kinetics of oxidation of As(III) adsorbed on iron oxide particles speeds up at pH = 9, but the process stops at a pH below 6 [15,16,17,18]. In the case of Se, the composition of water also has some impact on the oxidation equilibrium of Se species. The photo-oxidation of Se(IV) (as oxyanions and organo-derivatives) is significantly accelerated at a low pH, but could be inhibited by the presence of ions common in most water systems, such as chlorides [19]. Oxygenation of the water system may also cause changes in the ratio of Te oxyanions. Considering all the above, to prevent the speciation changes the sampling procedures should include filtration of the primary suspended particulate matter, and next the soluble forms of Te should be immediately separated.

Therefore, the crucial point of speciation analysis, especially in low concentrations, is maintaining the stability of the studied species. Sampling as the first step of each procedure could disrupt physicochemical equilibria between Te(IV) and Te(VI). Chemical fixation is not always the right answer; e.g., the addition of APDC to bind Te(IV) [20], followed by sedimentation of the inter-complex, could cause reduction of Te(VI) as an answer of the speciation system aimed to maintain the redox equilibrium in the studied water system [14]. Therefore, separation of chemical forms is a better solution than fixation [21,22]. One of proposed methods is separation based on retention of Te(IV) on γ-MPTMS (γ-Mercaptopropyltrimethoxysilane) modified silica-coated magnetic nanoparticles (SCMNPs) supported by ultrasounds. It offers a very low limit of detection (LOD) but the procedure has to be performed fully in the laboratory, and determination of Te(VI) requires oxidation (K_2_CrO_4_, pH = 0) of retained analyte (indirect analysis) [13].

Another approach is selective separation of speciation forms of elements during on-site sampling, using a simple methodology such as solid phase extraction (SPE). Due to its simplicity and transportable equipment, it can be performed immediately after collection of the samples. Such a procedure was proposed for separation of As, Se, Te, and Tl species [21,23,24,25,26]. For retention of As(III) and Te(IV) anions, a C18 SPE cartridge saturated with ammonium pyrrolidine dithiocarbamates was applied [23,26]. The cartridge was prepared in the laboratory and stored for 24 h before usage [23]. Also, cartridges filled with a strong anion exchange resin (SAX) were used to separate As(III) and As(V). Inorganic As(III) is neutral (pK_a_ = 9.2), and As(V) is in the form of an anion (pK_a_ = 2.3); therefore, only As(V) is retained on the column [21]. By analogy, and based on the literature data, separation of Te forms on the anionic column should be possible.

Usually, very low content of tellurium species could be detected with a combination of high-performance liquid chromatography, HPLC (mostly anion-exchange columns), and spectral detection [27]. Application of SPE and in sequence determination using graphite furnace atomic absorption spectrometry (GF-AAS) [28], also hyphenated with hydride generation (HG) [29] or inductively coupled plasma-mass spectrometry (ICP-MS) [20,26] allows the tellurium forms to be distinguished. Separation of the species just after sample collection, however, allows off-line determination and long-term storage.

Taking into account the above information and our own experience, we decided to perform separation of Te forms without prior interference with the primary speciation. A commercially available anion exchange SPE column was proposed to retain anionic Te(IV) and separate it from Te(VI), which can be leached later, not necessarily in the field. The proposed procedure was developed to be applied on a large scale in routine environmental monitoring of water to determine Te forms with simple and fast elemental techniques such as ICP-MS and/or inductively coupled plasma optical emission spectrometry (ICP-OES), after on-site sample pretreatment.

## 2. Results and Discussion

### 2.1. Stability Study of Te Speciation

Speciation analysis is not only a methodological issue, but also requires consideration of the stability of the equilibrium between dissolved forms (dissolved phase < 0.45 µm) and an effect of Fe and Mn oxide sedimentation after sampling (oxidation of water). The secondary sedimentation of Fe(III/II)Ox affects both forms of Te oxyanions, but Te(IV), unlike Te(VI), can be also adsorbed via the formation of surface complexes [30]. When it comes to MnOx, it is Te(VI) that has a much greater affinity, while Te(IV) can be oxidized on the Mn oxide surface prior to the sorption, and after that it acts as Te(VI) [31]. Another study shows that the MnOx phase is important in the immobilization of both Te forms, while FeOx adsorbs mostly Te(IV) [32]. These results, regardless of the outcome, indicate that separation of the suspension should be applied to restrain changes in Te speciation that result from sampling, and next the soluble forms of Te should be immediately separated.

For assessment of the stability of Te species, surface water from the Wkra River was used as the water matrix. The water sample was filtered, then spiked with Te (200 ng mL^−1^ of Te(IV) or/and 200 ng mL^−1^ of Te(VI)), and immediately analyzed using ICP-MS and HG ICP-OES. The samples were stored at 4 °C, and analyzed again several times. Obtained data showed that during three weeks of storage no sedimentation occurred in the sample for Te(IV) (both ICP-MS and HG ICP-OES results differed by less than 2%). For Te(VI), some small losses were observed already after one week (6% decrease detected using ICP-MS), and progressed in time (14% after three weeks). A similar experiment was performed for river water samples spiked with Te before filtration. Here, the changes in the concentration, calculated relative to the initial content, after 7 days of storage achieved 6% for Te(IV) and 21% for Te(VI), and after 10 days increased to 8% for Te(IV) and 28% for Te(VI).

### 2.2. Conditions for Separation of Te(IV) and Te(VI)

**Figure 1:**
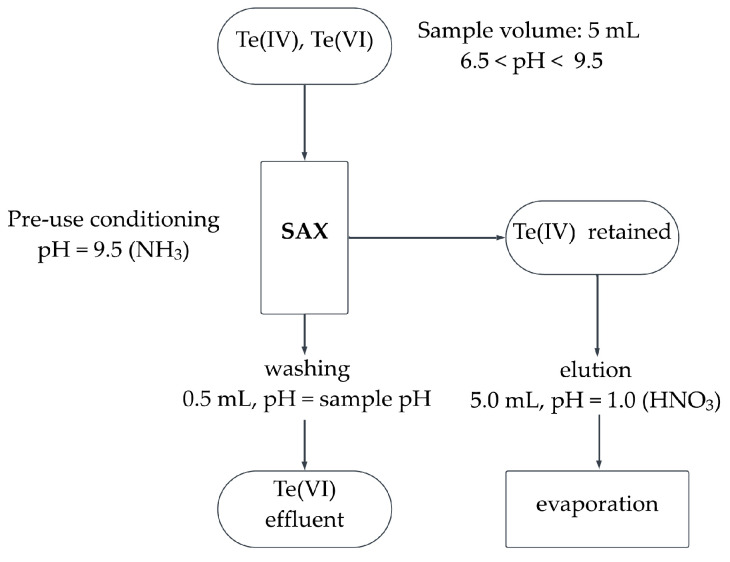
The scheme of the proposed separation procedure.

Application of anionic resin directly to the sampling site should be a solution to that problem and allow for rapid separation of Te(IV) and Te(VI). The anions differ in protonation, which depends on the pH of the solution. Based on the literature data, it was concluded that there is a pH at which one form of tellurium is protonated and the other remains an anion. Thanks to this, it is theoretically possible to separate a mixture of tellurite (IV) and tellurate (VI) on anion exchange columns. The protonated form should pass through the column with no interaction, and the anion would be retained. Next, washing the column with a solution of a suitably low pH should result in the protonation of the retained anionic form of Te, which will be eluted from the column. This would make it possible to directly determine individual forms of tellurium (Figure 1). The retention of all species of interest was examined by ICP-MS and HG-ICP-OES determination of Te in effluents obtained after the SPE procedure.

It has to be underlined that the literature data on dissociation constants of telluric acids are quite inconsistent: for H_2_TeO_3_, the reported values of pKa_1_ are in the range 2.46–7.74 and pKa_2_ 7.74–10.7, and for H_6_TeO_6_ the values are pKa_1_ 6.17–8.8 and pKa_2_ 10.09–11.19 [33]. Therefore, the choice of the optimal conditions for separation of both Te species was not obvious.

**Figure 2:**
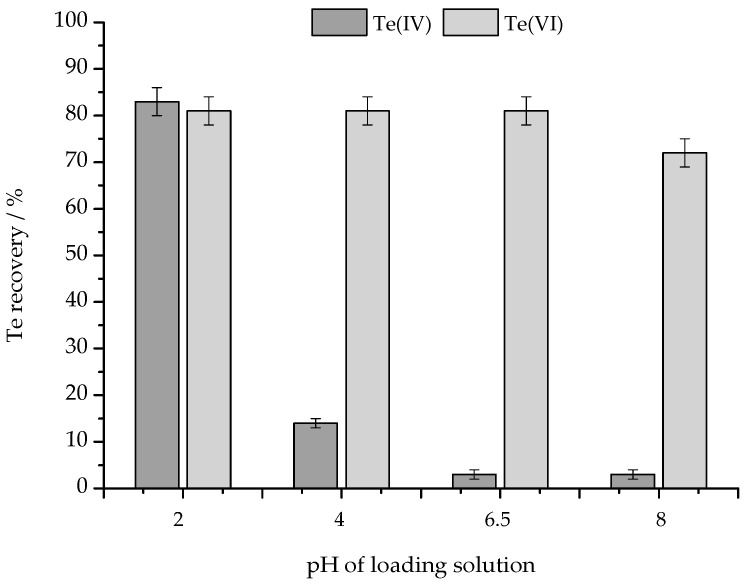
Recovery of Te in effluents from SAX columns after loading of 2.00 mL containing 100 ng of standard solutions of Te(IV) or Te(VI). The standards were adjusted to pH = 2.0, 4.0, 6.5 or 8.0.

The rate of protonation of tellurium oxyanions depends on the pH of the solution. The fully protonated form will not be retained on anion-exchange resin, which is the basis for the separation of Te(IV) and Te(VI). To find the optimal pH of the sample, tellurium standards were prepared in the pH range 2–8, and loaded to the SPE columns. The results are presented in Figure 2.

**Figure 3:**
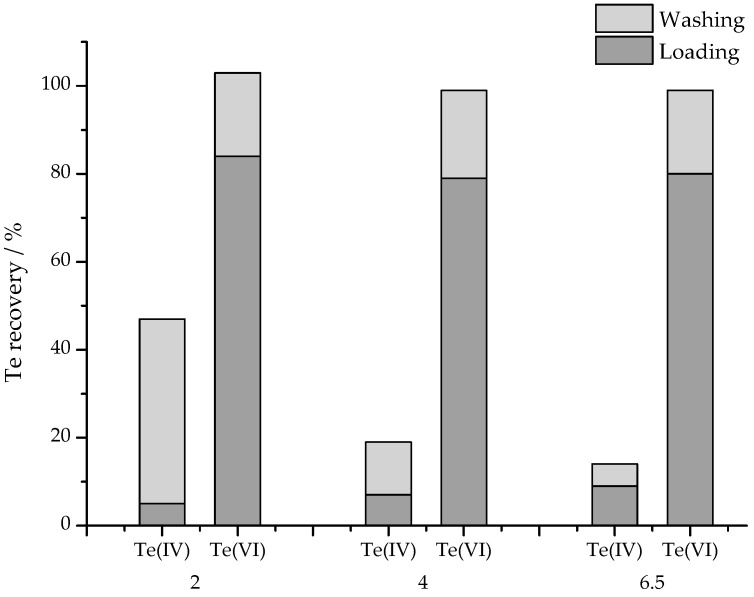
Recovery of Te in solutions obtained in SPE procedure. SAX columns were loaded with 2.00 mL containing 100 ng of standard solutions of Te(IV) or Te(VI), and then washed with 2.0 mL water solutions adjusted to pH equal to 2.0, 4.0, or 6.5 (RSD < 3%).

Te(IV) was successfully retained on the column when the pH of the solution was below 6.5. Te(VI) passed through the column regardless of pH, but at higher pH (<8.0) partial retention might be observed. The recovery of Te(VI) was around 80%, so in this case the washing step cannot be omitted. The next goal was to wash out the remaining Te(VI) without removing Te(IV). For this purpose, washing solutions with pH = 2.0, 4.0, and 6.5 were tested and compared (Figure 3).

Based on the results of ICP-MS determinations, it was found that in the tested range, the recovery of Te(VI) does not depend significantly on the pH of the washing solution. In each case, approximately 80% of Te(VI) passes through the sorbent without interacting with it (loading of the sample), and in the washing step, the remaining 20% is quantitatively eluted regardless of the pH of the eluent. In turn, for Te(IV), the recovery in the first step remains below 10%, while in the second step it increases with the acidity of the eluent, starting from 5% for pH = 6.5, and reaching 42% for pH = 2.0.

### 2.3. Optimization of the SPE Procedure

These preliminary data show that adjusting the pH of solutions passing through the SPE column should allow efficient separation of Te(IV) and Te(VI), but the procedure still needs to be optimized. When the pH is too low (lower than 6.5), retention of Te(IV) is less effective and part of this form is eluted from the column. With too high a pH, washing of Te(VI) might be affected. Further optimization of the separation procedure was focused on two aspects: minimization of Te(IV) losses during sample loading, and completeness of Te(VI) removal from the column. Several parameters were checked—pre-use conditioning of the SPE columns (different pH and solvents), pH of the sample, pH of the solution used for washing of the column after sample loading (removal of remaining Te(VI))—and the sample volume was increased to 10 mL (Table 1).

Loading a higher sample volume (10.0 mL) showed that retention of Te(IV) is not quantitative, and ca. 30% is removed from the column. Slightly lower losses were observed when the columns were conditioned in HNO_3_ (pH = 3) than in methanol. During the washing step, intended to remove Te(VI), Te(IV) is also gradually removed from the column. Washing of Te(VI), however, was successful. Just 1.0 mL of the washing solution is enough to completely remove it from the column. In the next step, complete elution of Te(IV) was achieved with 5.0 mL of HNO_3_ (pH = 1), which was more efficient than HNO_3_ (pH = 2), and thus pH 1 was chosen as optimal in further studies. For Te(VI), no significant differences were observed regardless of the conditioning conditions or the pH of the washing solution. This form is not retained on the column, and the small part remaining after sample loading can be removed with just 1.0 mL of a solution (pH = 6.5 and 7.5). Further experiments, which aimed at minimizing the losses of Te(IV) during the washing step, have shown that the volume of the washing solution can be decreased even to 0.5 mL.

**Figure 4:**
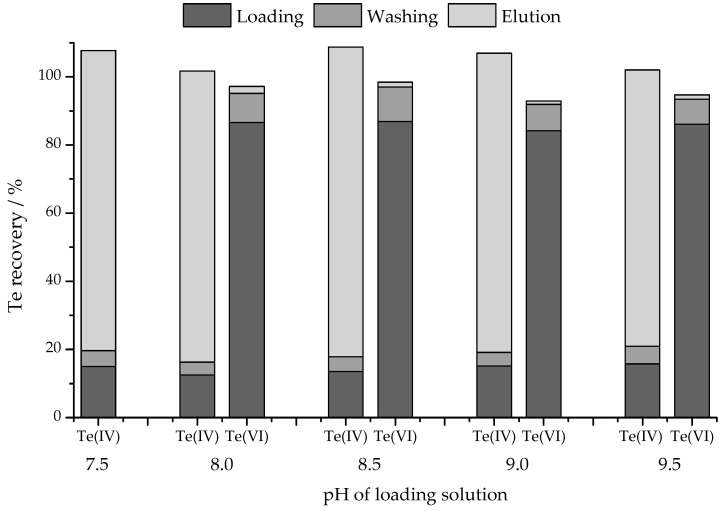
Recovery of Te from SAX columns. Conditioning: 4 mL (pH = 7.5). Loading: 5.00 mL with 100 ng of standard solutions of Te(IV) or Te(VI), 7.5 < pH < 9.5. Washing: 1.00 mL, pH = 7.5. Elution: 5.00 mL of HNO_3_ (pH = 1). (RSD < 3%).

The next experiments were designed to limit the losses of Te(IV) during the step of sample loading (Table 2). The results were compared for different conditions of column preparation and sample volume (2.00 and 5.00 mL). It was confirmed that in given conditions Te(IV) is not fully retained on the column during sample loading. The losses were higher for bigger sample volumes while the concentration of Te in the effluent remained constant. Retention of Te(IV) was slightly more efficient when the column was pre-conditioned at pH = 3 than pH = 2, and thus in the next experiments the pre-conditioning was performed with a solution with a pH above 7. Also, it was checked whether changes in the sample pH have an effect on the retention of both Te(IV) and Te(VI). The pre-conditioning of the columns was performed with a solution of pH = 7.5 (Figure 4) and 9.5 (Table 3), and the sample pH was adjusted in the range 7.5–9.5 (Figure 4 and Table 3). Pre-conditioning of the SPE columns in alkaline pH partially limits the losses of Te(IV), although a 10–15% loss should still be expected during the step of sample loading, and an additional 4–5% during washing. Changes in the pH do not significantly impact the behavior of Te(VI)—it is not retained regardless of the pH of the pre-conditioning solution or the sample, within the tested range.

### 2.4. Limit of Detection

The limit of detection (LOD) was calculated as a mean value increased by three times the standard deviation of the tellurium concentration in the blank sample ($\bar{x}$ + 3 SD, n = 6). The limit of quantification (LOQ) was calculated as a mean value increased by ten times the standard deviation of the tellurium concentration in the blank sample ($\bar{x}$ + 10 SD, n = 6). The obtained values (for a 5.0 mL sample) are as follows: LOD equals 0.02 ng mL^−1^ for Te(VI) (step 2 of the SPE procedure) and 0.05 ng mL^−1^ for Te(IV) (step 3), and LOQ equals 0.05 ng mL^−1^ for Te(VI) and 0.14 ng mL^−1^ for Te(IV). The natural Te content is much lower, but the proposed technique is to be used for research in contaminated areas and, above all, for research where the separation step is potentially to be carried out in the field.

Due to the limited sample volume (5 mL) and the necessity to use a similar volume of solution to elute Te(IV), the proposed procedure only allows for separation of both Te forms, but no preconcentration is obtained. This can be achieved by evaporation, which can be simply performed using a crucible covered with a watch glass. A total of 4.00 mL of 10 ng mL^−1^ Te(IV) or Te(VI) standard solution was evaporated almost to dryness on a hot plate. To avoid volatilization of Te compounds, the temperature during evaporation did not exceed 100 °C. The residue was dissolved in 2.00 mL of water. In such conditions, the recovery of Te(IV) standard was 84–101%, which confirms that no significant losses of the analyte occur.

A similar experiment was performed with solutions obtained in the SPE procedure. A mixture of standard solutions of both Te forms (5.00 mL containing 10 µg g^−1^ Te(IV) and 10 µg g^−1^ Te(VI)) was loaded to SAX column, and the column was washed with 0.50 mL solution pH = 7.5. The effluents were collected together and evaporated on a hot plate, and the residue was dissolved in 2.0 mL of water. Te(IV) was eluted from the column with 5.00 mL 0.1 mol L^−1^ HNO_3_, and this solution was also evaporated following the same procedure. The recoveries were 88.8 ± 1.2% for Te(VI), and 83.8 ± 5.2% for Te(IV), which gives a total recovery of 86.2 ± 2.4%. For comparison, the recoveries obtained in the SPE procedure without the evaporation step were 107.4 ± 2.6% for Te(VI), 88.6 ± 3.2% for Te(IV), and total recovery 97.9 ± 2.4%.

### 2.5. Interferences

Another important aspect are potential interferences from other anions present in water samples, mostly in a great excess compared to Te oxyanions. The anions of concern are nitrates, chlorides, and phosphates. It was shown that the separation step reduces the effect of a high content of chloride ions in seawater on determination [13,21]. To check the influence of other anions on separation of Te(IV) and Te(VI), a mixture of both Te standards was prepared in the presence of an excess of ammonium nitrate (model ion), and the SPE procedure was performed (Table 4). Determination of total Te in the initial solutions shows that the measurements are prone to positive errors, increasing with the increasing concentration of nitrates. However, after SPE, the results were correct (recovery in the range 98–104%). This demonstrates that the proposed SPE procedure is not only resistant to interference from nitrates (up to 4000 molar excess) but also that its application helps avoid errors during ICP-MS measurements.

### 2.6. Analytical Application

The final step of this study was utilization of the proposed procedure of separation of Te species. It was applied to determine Te(IV) and Te(VI) in two types of natural water matrix: river water and seawater. The sample pH was 8.2 for river water, and 6.8 for seawater. Originally, both water samples did not contain measurable amounts of Te. Therefore, they were spiked with both forms of tellurium, i.e., Te(IV) or/and Te(VI), to obtain the final concentration of 200 ng mL^−1^ for each form. The samples were then filtered through a 0.45 µm cellulose nitrate filter, subjected to the SPE procedure (as described in Section 3), and Te was determined with ICP-MS (total content). Additionally, Te(IV) was determined in obtained solutions using HG ICP-OES (Table 5). Such an approach allows any changes in speciation that may be occurring during the SPE procedure to be tracked. For both river water and seawater, when the samples were spiked with only Te(IV), the results obtained with both techniques were in a very good agreement. For samples spiked with only Te(VI), no Te was detected with HG ICP-OES. This proves that the SPE procedure causes no significant shifts between Te(IV) and Te(VI), even in the presence of diversified matrices. Separation of Te(IV) and Te(VI) was not hindered by the presence of a river water matrix—Te(VI) almost completely leaves the column during sample loading, and the amount of Te(IV) that is not retained in this step is similar to the results obtained for standard solutions. Separation is slightly less effective in the case of seawater—Te(VI) still leaves the column unaffected but only about 70% of Te(IV) is retained. Nevertheless, for both types of water samples, total recovery of Te from the SPE procedure was complete (96–101%). Thus, the proposed procedure allows for effective separation of Te(IV) and Te(VI) in the case of less loaded water matrices, such as river water. In the case of more complex samples, such as seawater containing significant quantities of chlorides and other ions, as well as pollutants resulting from the harbour activities, the application of SAX columns additionally eliminates matrix compounds, which otherwise lead to positive errors during Te determination. For such difficult samples, it will be beneficial to use SPE as the sample preparation method to eliminate matrix components causing interferences, and then both detection techniques (ICP-MS and HG ICP-OES) to obtain complementary information on Te total content and speciation.

Considering the potential application of the proposed method at the sampling site, the possibility of storing the SPE columns with retained Te(IV) and its postponed elution were tested. The recovery study was conducted based on a river water matrix (Wkra). The elution after one day of storage was equally as effective as immediate elution, and resulted in 100% recovery of Te(IV). After 7 days of storage, the recovery was only 66% (compared to leaching at the time of application). This indicates that it is not necessary to elute Te(IV) at the sampling site; elution can be carried out after transportation to the lab. Prolonged storage of the columns, however, is not recommended.

## 3. Materials and Methods

The reagents used were 65% HNO_3_ (d = 1.40 g mL^−1^) (Supelco, Merck KGaA, Darmstadt, Germany), 25% NH_3_ (Chempur, Poland), K_2_TeO_3_ (M = 253.79 g mol^−1^) (Sigma-Aldrich, Merck KGaA, Germany), K_2_TeO_4_·H_2_O (M = 287.79 g mol^−1^) (Sigma-Aldrich, Merck KGaA, Germany), ICP multi-element standard solution VIII Certipur (100 mg L^−1^: Al, B, Ba, Be, Bi, Ca, Cd, Co, Cr, Cu, Fe, Ga, K, Li, Mg, Mn, Na, Ni, Pb, Se, Sr, Te, Tl, Zn in dilute HNO_3_) (Supelco, Merck KGaA, Germany). Standard solutions of Te(IV) and Te(VI) were prepared by dissolving, respectively, 0.0208 g K_2_TeO_3_ and 0.0248 g K_2_TeO_4_·xH_2_O in 100.0 mL deionized (DI) water, and further dilution with DI water to obtain an appropriate concentration. Intermethod comparison (ICP-MS and HG-ICP-OES) was used to control the stability of the standards. Optionally, the pH of the standards was adjusted to 2, 4 or 6.5 by adding 1 mol L^−1^ HNO_3_ or 25% ammonia. The eluting solutions of fixed pH (2, 4 and 6.5) were prepared by adding 1 mol L^−1^ HNO_3_ or 25% ammonia to deionized water until the required pH was reached. The pH was controlled by pH Meter Seven Direct SD20 (Mettler Toledo, Greifensee, Switzerland). Deionized water was obtained from Arium Mini Plus Ultrapure Water System (Sartorius, Göttingen, Germany).

For separation, an Agilent SampliQ 12-Positions solid phase extraction chamber (Agilent, Santa Clara, CA, USA) connected with a vacuum pump (KNF, Freiburg, Germany) was used, together with SAX columns (3 mL) (trimethylaminopropyl, strong anion—Quaternary amine Bond Elut, Agilent, USA), where typical carbon loading is 10.9%, surface area 500 m^2^ g^−1^, particle size 40 µm, and mean pore size 60 Å.

For analytical application, surface water from the Wkra River (a small river located 43 km north-west of Warsaw, Poland) and water from the Baltic Sea sampled in the harbour of Darłowo (Poland) were used as the water matrix. The water sample contained a small quantity of surfactants. The suspended particulate matter (SPM) content was negligible (40 µg mL^−1^), and Te undetectable (<LOD = 0.002 ng mL^−1^). To remove SPM, sample separation included vacuum filtration (0.45 µm). The SPM content was estimated after drying and weighing of the residue.

The proposed separation procedure (Figure 1) includes the following steps: Sorbent cleaning and conditioning with 4.0 mL of NH_3_ solution of pH = 9.5; loading of 2.0–5.0 mL of water sample and collection of the effluents; washing with 0.5 mL solution of pH similar to the pH of the sample (6.5 < pH < 9.5); leaching of Te retained on the sorbent with 5.0 mL of 0.1 mol L^−1^ HNO_3_. Flow rate was equal to 1.3 mL min^−1^ and each column was used once. If necessary, the volume of the analyzed sample can be reduced by evaporation (temp. < 100 °C).

For determination of Te, a NEXION 300D mass spectrometer (PerkinElmer, Shelton, CT, USA) was used. ICP-MS determinations were performed with the following parameters: sweep: 40, repetitions: 5, dwell time: 50 ms, ICP RF power: 1300 W, deflector voltage: −11.75 V, nebulizer gas flow: 0.82 L min^−1^, plasma gas flow: 16 L min^−1^, auxiliary gas flow: 1.575 L min^−1^ and monitored isotopes: ^125^Te, ^126^Te, ^128^Te. The calibration curve method was used. The standard solution was analyzed after every 10 samples to control the instrumental drift.

For determination of Te(IV), an Agilent 5800 optical emission spectrometer (ICP-OES) (Agilent Technologies, Penang, Malaysia) equipped with the Multi-Mode Sample Introduction System (MSIS) accessory was used. The MSIS was used as a hydride generation (HG) system. The reductant solution contained 1% NaBH_4_ (*w*/*v*) in 0.1% NaOH (*w*/*v*), where NaBH_4_ acted as the reducing agent to generate the gaseous tellurium hydride, and NaOH was used as a stabilizer. The upper inlet of the MSIS unit was used to provide a reductant solution. The lower inlet delivered a sample solution prepared in 2 mol L^−1^ HCl. A nebulizer sample channel stayed blocked in HG mode. All reagents were pumped continuously to the MSIS with the peristaltic pump speed set at 12 rpm (0.75 mL min^−1^), and mixed at the tops of the inlets. Volatile hydrides were formed and carried with gas from the MSIS unit to ICP torch. The axial torch view was used in this work. RF power was set up at 1450 W. Plasma, nebulizer, and auxiliary argon flow rates were 12 L min^−1^, 0.7 L min^−1^ and 1.0 L min^−1^, respectively. Two analytical wavelengths were monitored: 214.282 and 238.579 nm. An external calibration curve method was used. Single-element working standards were treated in the same manner as the samples and prepared in 2 mol L^−1^ HCl solution.

## 4. Conclusions

The development of the photovoltaic industry raises concerns that solar panels containing CdTe might become a source of Te anions in water systems. Te(IV) and Te(VI) differ in mobility, retention on suspended matter, and toxicity, and thus speciation analysis should be implemented in the monitoring of potentially polluted sites. In the case of speciation analysis, the moment of sample collection, decisions about its preservation, and stability during transport are of crucial importance. On-site separation of Te(IV) and Te(VI) using solid phase extraction is a simple way to minimize the preliminary speciation changes. Having a simple separation procedure (without specially modified SPE columns) is important from the point of view of water monitoring in the context of Te speciation. The SPE procedure based on SAX columns resulted in almost total recovery of Te(VI). For Te(IV), a 10–15% loss was observed during the step of sample loading, and up to 5% during washing. Commercially available SAX columns, just conditioned with an alkaline solution (pH = 9.5), can be used with samples of pH in the range of 6.5–9.5, containing up to 4 000 molar excess of nitrates. This covers the range of pH and anion content that can be expected in natural waters. The limit of detection is 0.02 ng mL^−1^ for Te(VI) and 0.05 ng mL^−1^ for Te(IV). After simple reduction of the sample volume by evaporation, even lower values (pg mL^−1^) can be achieved, which are sufficient for tellurium determination in natural water samples. The proposed procedure could be applied on a large scale in routine environmental monitoring of water to determine Te forms with elemental techniques such as ICP-MS, after on-site sample pretreatment. Separation of the species just after sample collection allows off-line determination and long-term storage. Also, there is no need for leaching out the retained form at the sampling site, which simplifies the operation of sample collection, shortens time in the field to less than 10 min per sample (when taking multiple samples from one water reservoir, up to 24 samples can be separated simultaneously), and restricts errors.

## Figures and Tables

**Table 1:** Recovery [%] of Te from SAX columns. Conditioning: 2 mL of either HNO_3_ (pH = 3) or methanol, then 2 mL of H_2_O. Loading: 10.0 mL with 100 ng of standard solution of Te(IV) or Te(VI), pH = 6.5 or 7.5. Washing: 3 portions of 1.00 mL, pH = 6.5 or 7.5. Elution: 5.00 mL of HNO_3_ (pH = 1 or 2) in two portions (3.00 mL, then 2.00 mL).

**Conditioning**	**pH = 3**	**MeOH**	**pH = 3**	**MeOH**	**pH = 3**	**MeOH**	**pH = 3**	**MeOH**
**Loading**	**Te(IV) pH = 6.5**				**Te(VI) pH = 7.5**		**Te(VI) pH = 6.5**	
10 mL	26 ± 1	33 ± 1	27 ± 1	34 ± 1	89 ± 2	100 ± 3	95 ± 2	98 ± 2
**Washing**	**pH = 6.5**				**pH = 7.5**		**pH = 6.5**	
1 mL	3.1 ± 0.1	3.4 ± 0.1	3.4 ± 0.1	3.5 ± 0.1	3.3 ± 0.1	4.2 ± 0.1	3.2 ± 0.1	3.4 ± 0.1
1 mL	3.1 ± 0.1	3.0 ± 0.1	3.0 ± 0.1	1.1 ± 0.1	0.5 ± 0.1	0.5 ± 0.1	0.1 ± 0.1	0.1 ± 0.1
1 mL	3.2 ± 0.1	3.6 ± 0.1	3.3 ± 0.2	3.5 ± 0.1	<0.1	<0.1	<0.1	<0.1
Total (3 mL)	9.4 ± 0.2	9.9 ± 0.2	9.6 ± 0.2	8.1 ± 0.2	3.9 ± 0.1	4.7 ± 0.1	3.4 ± 0.1	3.5 ± 0.1
**Elution**	**pH = 1**		**pH = 2**		**pH = 2**		**pH = 2**	
3 mL	56.2 ± 1.3	55.5 ± 0.7	31.5 ± 0.9	31.3 ± 1.0	<0.1	<0.1	<0.1	<0.1
2 mL	4.6 ± 0.2	8.2 ± 0.2	12.1 ± 0.2	13.7 ± 0.6	<0.1	<0.1	<0.1	<0.1
Total (5 mL)	60.8 ± 1.3	63.7 ± 0.7	43.7 ± 0.9	45.0 ± 1.2	<0.2	<0.2	<0.2	<0.2
**Total recovery**	96 ± 2	107 ± 2	80 ± 2	87 ± 2	93 ± 2	105 ± 3	98 ± 2	102 ± 2

**Table 2:** Recovery [%] of Te from SAX columns. Conditioning: 2 mL of HNO_3_ (pH = 2 or 3), then 2 mL of H_2_O. Loading: 2.00 mL with 100 ng or 5.00 mL with 100 ng of standard solutions of Te(IV), pH = 6.5. Washing: 1.00 mL, pH = 6.5. Elution: 5.00 mL of HNO_3_ (pH = 1).

Conditioning	pH = 2		pH = 3	
Sample Volume	2.00 mL	5.00 mL	2.00 mL	5.00 mL
Effluent	8.7 ± 0.3	15.2 ± 0.6	5.0 ± 0.2	14.5 ± 0.6
Washing	8.0 ± 0.3	4.9 ± 0.1	5.0 ± 0.2	4.5 ± 0.1
Elution	89.1 ± 1.6	88.9 ± 2.2	97.6 ± 2.6	92.0 ± 2.6
Total	105.8 ± 1.7	109.0 ± 2.3	107.6 ± 2.6	111.0 ± 2.6

**Table 3:** Recovery [%] of Te from SAX columns. Conditioning: 4 mL (pH = 9.5). Loading: 5.00 mL of 20 µg g^−1^ standard solution of Te(IV) or Te(VI) (100 ng), pH = 7.5 or 9.5. Washing: 1.00 mL, pH = 7.5 or 9.5 (corresponding to the pH of the sample). Elution: 5.00 mL of HNO_3_ (pH = 1).

	Te(IV)		Te(VI)	
Sample pH	7.5	9.5	7.5	9.5
Effluent	10.8 ± 0.5	13.2 ± 0.4	85.8 ± 1.3	85.6 ± 2.2
Washing	4.8 ± 0.1	4.7 ± 0.2	8.2 ± 0.1	7.8 ± 0.2
Elution	78.9 ± 1.7	82.2 ± 1.3	0.9 ± 0.1	1.4 ± 0.1
Total	94.4 ± 1.8	100.1 ± 1.4	94.8 ± 1.3	94.9 ± 2.2

**Table 4:** The amount of Te [ng] determined in solutions obtained during the SPE procedure using SAX columns, and Te recovery [%] (in brackets). Conditioning: 4 mL (pH = 9.5). Loading: 5.00 mL of solution containing Te(IV) and Te(VI) (125 ng of each form) in the presence of 4, 40 or 400 mg L^−1^ NH_4_NO_3_, pH = 7.5. Washing: 1.00 mL, pH = 7.5. Elution: 5.00 mL of HNO_3_ (pH = 1).

Molar Excess of NO_3_^−^	40	400	4000
Loading + Washing	123 ± 3 (49.3 ± 1.0%)	121 ± 2 (48.5 ± 0.7%)	128 ± 2 (51.4 ± 1.0%)
Elution	136 ± 3 (54.3 ± 1.0%)	124 ± 4 (49.5 ± 1.5%)	124 ± 3 (49.5 ± 1.1%)
Total	259 ± 4 (104 ± 1.4%)	245 ± 5 (98.0 ± 1.7%)	252 ± 4 (101 ± 1.5%)
Initial solution	260 ± 8 (104 ± 3%)	272 ± 7 (109 ± 3%)	293 ± 8 (117 ± 3%)

**Table 5:** Recovery [%] of Te from water samples spiked with 200 ng mL^−1^ Te(IV) or/and Te(VI). The samples were subjected to the SPE procedure using SAX columns, and Te was determined with ICP-MS (total) and HG ICP-OES (Te(IV)). The recovery was calculated in relation to the total Te content. (* LOD = 0.2 ng mL^−1^).

Sample		River Water		Seawater	
		ICP-MS	HG ICP-OES	ICP-MS	HG ICP-OES
Te(IV)	Loading + Washing	18.2 ± 0.5	18.5 ± 0.1	29.6 ± 0.6	28.0 ± 0.5
	Elution	82.3 ± 2.5	77.4 ± 0.4	68.2 ± 1.3	70.7 ± 0.7
	Total	100.5 ± 2.6	95.9 ± 0.5	97.8 ± 1.5	98.7 ± 0.9
Te(VI)	Loading + Washing	91.4 ± 2.7	<LOD *	97.4 ± 1.4	<LOD
	Elution	4.8 ± 0.1	<LOD *	2.5 ± 0.2	<LOD
	Total	96.2 ± 2.7	<LOD *	99.8 ± 1.5	<LOD
Te(IV) + Te(VI)	Loading + Washing	58.7 ± 1.7	9.1 ± 0.1	61.5 ± 1.8	16.3 ± 0.2
	Elution	42.3 ± 1.2	38.7 ± 0.2	34.0 ± 0.5	34.2 ± 0.1
	Total	101.0 ± 2.1	47.8 ± 0.3	95.5 ± 1.9	50.5 ± 0.3

## Data Availability

Dataset available on request from the authors.
